# Dopamine in Fear Extinction

**DOI:** 10.3389/fnsyn.2021.635879

**Published:** 2021-03-01

**Authors:** Ximena I. Salinas-Hernández, Sevil Duvarci

**Affiliations:** Institute of Neurophysiology, Neuroscience Center, Goethe University, Frankfurt, Germany

**Keywords:** dopamine, fear extinction, amygdala, medial prefrontal cortex, nucleus accumbens

## Abstract

The ability to extinguish fear memories when threats are no longer present is critical for adaptive behavior. Fear extinction represents a new learning process that eventually leads to the formation of extinction memories. Understanding the neural basis of fear extinction has considerable clinical significance as deficits in extinction learning are the hallmark of human anxiety disorders. In recent years, the dopamine (DA) system has emerged as one of the key regulators of fear extinction. In this review article, we highlight recent advances that have demonstrated the crucial role DA plays in mediating different phases of fear extinction. Emerging concepts and outstanding questions for future research are also discussed.

## Introduction

Learning to associate stimuli and situations with danger or safety is critical for survival and adaptive behavior. In the laboratory, these forms of learning are typically studied using Pavlovian fear conditioning and extinction. Fear conditioning is an example of associative learning in which an initially neutral stimulus such as a tone (conditioned stimulus, CS) comes to elicit fear responses after being paired in time with an aversive outcome such as a foot shock (unconditioned stimulus, US). Once the CS-US association is learned, subsequently repeated presentations of the CS in the absence of the aversive US result in a gradual decrease in conditioned fear responses, a process known as fear extinction. In the last decades, fear extinction has attracted much interest in part because deficits in extinction learning are thought to underlie human anxiety disorders, such as post-traumatic stress disorder (PTSD) and phobias (Graham and Milad, 2011; Pitman et al., 2012; Craske et al., 2017), and thus, understanding the neural basis of fear extinction has high clinical significance. Decades of research has revealed that a distributed network of brain structures including mainly the amygdala and the medial prefrontal cortex (mPFC) mediates the acquisition and consolidation of fear extinction memories (Pape and Pare, 2010; Sotres-Bayon and Quirk, 2010; Maren et al., 2013; Duvarci and Pare, 2014; Tovote et al., 2015).

In recent years, the dopamine (DA) system has also emerged as an important mediator of fear extinction. DA is a neurotransmitter critically involved in a wide range of functions including reward learning, motivation, motor control, and cognitive functioning. DA neurons that are mainly located in the ventral tegmental area (VTA) and substantia nigra (SN) in the midbrain. DA receptors are metabotropic receptors that can be classified into two main types with the DA D1-type receptors (Gs-coupled) comprised of D1 and D5 and the DA D2-type receptors (Gi-coupled) comprised of D2, D3, and D4 subtypes (Missale et al., 1998). Systemic administration of DA precursor L-DOPA or D1-type receptor agonists before or right after extinction enhances acquisition and retention of fear extinction, respectively (Haaker et al., 2013; Abraham et al., 2016; Whittle et al., 2016), indicating the involvement of DA during both acquisition and consolidation of extinction memories. In particular, DA neurons located in the VTA, through their projections to the structures involved in fear extinction such as the amygdala and mPFC, are implicated in fear extinction. In this review, we highlight recent findings that have revealed the role DA plays in mediating different phases of fear extinction. We focus the discussion on the extinction of cued fear conditioning in rodents where recent progress has been made. We begin by discussing how fear extinction learning is initiated and driven by the activity of VTA DA neurons. We also discuss the emerging concept that fear extinction may be an appetitive learning process mediated by the brain’s reward circuitry. We next focus on how DA regulates the acquisition and expression of fear extinction in the amygdala. We consider the possible targets in the amygdala microcircuitry that DA can act on to mediate fear extinction. Finally, we discuss the role of DA in the mPFC in mediating the consolidation of extinction memories.

## A Dopamine Prediction Error Signal Initiates Fear Extinction Learning

Considerable evidence indicates that fear extinction represents new learning rather than forgetting or erasure of the original fear memory (Bouton et al., 2006; Myers and Davis, 2007). During extinction, the animal learns the new association between the presence of the CS and an unexpected safe outcome (i.e., the absence of the expected aversive US). Classical theories of associative learning postulate that learning is initiated by prediction errors (PE) that signal the discrepancy between expected and actual outcomes (Rescorla and Wagner, 1972) and new learning happens when outcomes do not match predictions. In fear extinction, the unexpected omission of the US induces a PE signal that leads to an update in the prediction associated with the CS so that it comes to be recognized as signaling safety. This in turn leads to a decay of conditioned fear responses.

**Figure 1:**
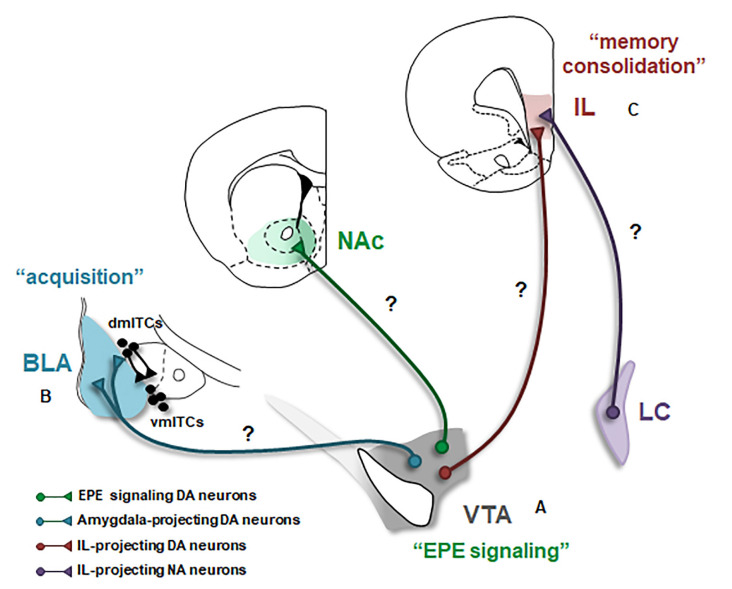
Dopaminergic modulation of the neural circuitry underlying fear extinction. Schematic of the three major projections of the ventral tegmental area (VTA) dopamine (DA) neurons likely involved in fear extinction are shown. BLA, basolateral amygdala; EPE, extinction prediction error; IL, infralimbic cortex; ITCs, intercalated cell masses (dm: dorsomedial, vm: ventromedial); LC, locus coeruleus; NA, noradrenalin; NAc, nucleus accumbens. Question marks (?) indicate possible DAergic projections mediating fear extinction. The involvement of these projections in extinction remains to be tested. **(A)** A subset of VTA DA neurons encodes an EPE signal that is necessary to initiate fear extinction learning. The projection target of EPE encoding DA neurons is currently unknown. NAc constitutes an ideal candidate however the exact subregion of NAc receiving the EPE signal remains to be determined. **(B)** Activation of DA receptors in the BLA mediates the acquisition of fear extinction memories. However, the source of DA input to the amygdala during extinction has not directly been demonstrated. Whether VTA DA projections to the BLA and also likely to dmITCs, are involved in fear extinction is an important outstanding question. **(C)** DA is crucial for the consolidation of extinction memories in the IL. The source of DA input to IL during fear extinction has remained elusive, however, IL receives its main DA input from the VTA and IL-projecting VTA DA neurons are thus plausible candidates. However, recent studies suggest that this DA projection is pro-aversive; and thus, DA released from other sources, such as NA neurons located in the LC might be more likely to mediate fear extinction.

Because not receiving an expected aversive US may be experienced as a rewarding event, the prediction error caused by the US omission during extinction (extinction prediction error, EPE) could be conceptualized as an appetitive or reward-like prediction error. Thus, fear extinction may be mediated by the reward learning system (Abraham et al., 2014; Josselyn and Frankland, 2018; Kalisch et al., 2019). It is well established that midbrain DA neurons encode reward prediction error (RPE) signals to drive reward learning (Schultz, 2006). Consistently, recent studies demonstrate that a subset of DA neurons, located in the VTA, is activated by the omission of the aversive US during fear extinction, and this increased DA neuron firing is indeed necessary to initiate fear extinction learning (Luo et al., 2018; Salinas-Hernández et al., 2018). Importantly, the timing of this DA signal fulfills the requirements of a prediction error: (i) it is specific to the time of the US omission; and (ii) it is observed during the early, but not late, trials of extinction learning indicating that it occurs specifically when the US omission is unexpected (Salinas-Hernández et al., 2018). Interestingly, a more recent study has further shown that although DA neurons located in both the medial and the lateral VTA, but not the SN, are activated by the omission of the US, particularly the medial VTA DA neurons encode an EPE signal to drive fear extinction. On the other hand, DA neurons that are found in the lateral VTA signal salience but not EPE (Cai et al., 2020). Together, these studies demonstrate that a PE signal encoded by a subset of DA neurons in the medial VTA is crucial to initiate and drive fear extinction learning (Figure 1A). If the PE encoded during fear extinction is an RPE signal, it is expected that the same DA neurons and DAergic circuits mediate these two signals. In support of this hypothesis, a recent study in fruit flies has shown that fear extinction is driven by the same distinct population of DA neurons that also mediates reward, but not fear, learning (Felsenberg et al., 2018). Whether in mammals the same DA neurons encode extinction and reward PE signals and whether these two distinct signals share similar properties are important questions for future research.

How and through which neural circuits this DA signal initiates extinction learning and ultimately leads to the plasticity underlying formation of extinction memories is currently unknown. The first step in addressing these questions is identifying the projection target of DA neurons that encode the EPE signal. Since DA neurons projecting to the nucleus accumbens (NAc), the main DA output region in the rodent ventral striatum, form the canonical reward circuitry (Wise, 2002), NAc constitutes a good candidate to fulfill this role (Figure 1A). Supporting this, an increase in DA release during fear extinction has been observed in the NAc (Badrinarayan et al., 2012) and the pharmacological blockade of DA receptors in the NAc impairs fear extinction learning (Holtzman-Assif et al., 2010). Furthermore, fear extinction learning in humans is accompanied by a prediction error-like activation in the ventral striatum (Raczka et al., 2011). However, in contrast with these findings, inhibition of DA terminals in NAc at the time of the US omission surprisingly does not affect extinction learning, although it does impair consolidation of extinction memory (Luo et al., 2018). Notably, single-unit recordings demonstrate that a small subpopulation of DA neurons mediate the EPE signal (Salinas-Hernández et al., 2018). It is therefore possible that this subpopulation of DA neurons projects to a specific and restricted subregion of NAc that was not targeted by Luo et al. (2018). In addition to NAc, other possible candidates include DA neurons projecting to the amygdala and/or mPFC. However, at odds with these possibilities, inhibition of DA terminals in the amygdala or mPFC during EPE signaling does not impair extinction learning (Luo et al., 2018). How a DA PE signal initiates extinction learning and leads to the acquisition and consolidation of extinction memories within the fear extinction circuitry is one of the key questions towards understanding the neural basis of fear extinction. Future research determining the exact projection target of EPE encoding DA neurons will be an important step in addressing this question (Figure 1A).

## Dopamine in The Amygdala Mediates The Acquisition of Fear Extinction Memories

The amygdala is a key structure underlying the acquisition and expression of fear extinction memories. Specifically, two subregions within the amygdala microcircuitry, the basolateral amygdala (BLA) and the intercalated cell masses (ITCs) are critically involved in fear extinction (Herry et al., 2010; Duvarci and Pare, 2014). Much evidence indicates that the BLA, consisting of the lateral and basal nuclei, is particularly required for the acquisition of extinction memories (Herry et al., 2008; Amano et al., 2011; Sierra-Mercado et al., 2011). A subpopulation of BLA neurons termed “extinction neurons” increases their firing to the CS during fear extinction, specifically late in the extinction session right before the animals show a decrease in conditioned fear responses (Herry et al., 2008; Amano et al., 2011). Furthermore, considerable evidence implicates GABAergic inhibition in the BLA during fear extinction. Both strengthening and also weakening of GABAergic transmission has been shown in the BLA during extinction (Marsicano et al., 2002; Chhatwal et al., 2005; Heldt and Ressler, 2007; Sangha et al., 2009; Kasugai et al., 2019), suggesting that particular subtypes of GABAergic interneurons are likely differentially recruited during extinction (Duvarci and Pare, 2014; Krabbe et al., 2018). However, the involvement of different subtypes of BLA interneurons in extinction has remained elusive. Importantly, GABAergic neurons are critical targets of DA in the amygdala and DAergic signaling has been demonstrated to suppress feedforward inhibition onto principal BLA neurons and facilitate synaptic plasticity in the BLA through activation of both D1 and D2 receptors (Bissière et al., 2003; Marowsky et al., 2005). For more detailed information on how DA regulates activity in the amygdala circuitry, the reader is referred to prior reviews (Abraham et al., 2014; Lee et al., 2017). Pharmacological studies show that blockade of both D1 and D2 receptors in the BLA impairs fear extinction. Notably, DA receptor antagonism in the BLA selectively affects the acquisition, but not consolidation, of fear extinction memories (Hikind and Maroun, 2008; Shi et al., 2017). Whether DA exerts its effects on fear extinction in the BLA through regulation of GABAergic interneurons is a critical question for further research. Furthermore, it will also be important to determine whether and how DA controls the activity of different subtypes of GABAergic interneurons in the BLA during extinction.

While pharmacological studies indicate the important role DA plays in the BLA during fear extinction, exactly how DA modulates extinction-related neuronal activity is not well understood. Research investigating reward learning in the BLA may provide some clues. Indeed, recent studies demonstrate that a genetically distinct and projection-defined subpopulation of BLA neurons mediates reward learning (Namburi et al., 2015; Kim et al., 2016). Intriguingly, supporting the hypothesis that fear extinction is an appetitive learning process, “reward neurons” overlap with “extinction neurons” in the BLA and these two types of neurons are functionally interchangeable (Zhang et al., 2020), suggesting that reward learning and fear extinction are indeed mediated by the same population of neurons in the BLA. Thus, studies investigating the role of DA in BLA during reward learning can provide valuable insights. In keeping with this, a recent study showed that VTA DA terminals in the BLA are activated during reward learning. Specifically, these terminals are activated by rewards and also reward-predicting CSs (Lutas et al., 2019). Whether VTA DA neurons projecting to BLA are also activated by CSs following fear extinction and whether DA input is critical for the CS responsiveness of “extinction neurons” will be key questions to address for future studies (Figure 1B).

As mentioned earlier, the second component of the amygdala microcircuitry crucial for fear extinction is the intercalated cell masses (ITCs), which are a network of interconnected GABAergic cell groups located in the external and intermediate capsules surrounding the BLA. Notably, the ITCs located within the intermediate capsule are comprised of the dorsomedial (dmITCs) and ventromedial (vmITCs) clusters where dmITCs exert a unidirectional inhibitory control over vmITCs (Paré et al., 2003; Ehrlich et al., 2009). Mounting evidence indicates that particularly the vmITCs, located between the BLA and the centromedial nucleus of the amygdala (CeM) are necessary for acquisition and expression of fear extinction memories (Jüngling et al., 2008; Likhtik et al., 2008; Amano et al., 2010; Busti et al., 2011). CeM constitutes the main output station of the amygdala necessary for fear expression (Ciocchi et al., 2010); and hence, vmITCs are indeed in an ideal position to suppress the expression of fear responses during fear extinction. They receive excitatory input from BLA and send inhibitory projections to CeM, and thus mediate feedforward inhibition of CeM (Paré et al., 2003; Mańko et al., 2011; Gregoriou et al., 2019). Anatomical studies show that D1 receptors are abundantly expressed in the ITCs (Jacobsen et al., 2006), suggesting an important role for DA in regulating the activity of these neurons. D1 receptors are typically Gs-coupled receptors and when activated they are expected to function in an excitatory fashion (Missale et al., 1998). Interestingly, D1 receptor signaling is unusual in ITCs, that is, DA through activation of D1 receptors hyperpolarizes ITCs and thus inhibits these neurons (Marowsky et al., 2005; Mańko et al., 2011). Furthermore, a recent study shows that the ITCs within the vmITC cluster are likely connected (Gregoriou et al., 2019; but also see Mańko et al., 2011) and activation of D1 receptors inhibits these local connections suggesting a general reduction in the output of the vmITCs (Gregoriou et al., 2019). Together, these studies indicate that dopamine inhibits the activity of ITCs and hence reduces the output of these neurons. Dopamine is therefore anticipated to reduce vmITC mediated feedforward inhibition of CeM. Because vmITCs are expected to be excited during extinction, whether DA plays a role in regulating the activity of vmITCs to mediate fear extinction is questionable. One plausible scenario is that DA might regulate the activity of vmITCs indirectly by inhibiting dmITCs and thereby disinhibiting vmITCs during extinction. According to this scenario, DA neurons are expected to differentially innervate and modulate these two distinct clusters of ITCs (Figure 1B). It will therefore be important for future studies to investigate whether and how DA input regulates the activity in distinct ITC clusters during fear extinction.

## Dopamine in The Medial Prefrontal Cortex Mediates Consolidation of Fear Extinction Memories

The mPFC, in particular the infra-limbic (IL) subregion of mPFC, is crucial for the consolidation of fear extinction memories (Sotres-Bayon and Quirk, 2010). Since dopaminergic signaling enhances signal-to-noise ratios and modulates synaptic plasticity in the mPFC (Seamans and Yang, 2004; Weele et al., 2019), DA is expected to play a vital role in the formation of extinction memories. Consistent with this, DA levels increase in the mPFC during fear extinction and remain elevated following extinction learning (Hugues et al., 2007). Conversely, selective ablation of mPFC-projecting catecholaminergic neurons has been found to impair retention of extinction (Morrow et al., 1999; Fernandez Espejo, 2003). Supporting these earlier findings, pharmacological studies have further revealed the role of prefrontal DA receptors in fear extinction. Administration of a D4 receptor antagonist in the IL following extinction impairs extinction retention the next day (Pfeiffer and Fendt, 2006). In addition, studies investigating the role of D1 and D2 receptors have found that inhibition of both D1 and D2 receptors in the IL during extinction learning impairs retention of extinction while acquisition remains intact (Hikind and Maroun, 2008; Mueller et al., 2010). Together, these findings suggest that activation of both D1- and D2-type receptors are required for consolidation, but not acquisition, of fear extinction memories in the IL.

Long-lasting plastic changes in the activity of IL neurons underlie extinction memories (Sotres-Bayon and Quirk, 2010). However, how different DA receptors contribute to the extinction-related changes in IL neurons is not yet well understood. Studies combining pharmacological manipulations with electrophysiology can provide some clues. Notably, IL neurons exhibit increased firing to the CS during retention of extinction (Milad and Quirk, 2002) and administration of a D2 receptor antagonist in the IL has been shown to attenuate these extinction-related CS responses (Mueller et al., 2010). Furthermore, consolidation of extinction memories requires NMDA receptor-dependent burst firing of IL neurons shortly after extinction learning, that is, during the period when memory consolidation takes place (Burgos-Robles et al., 2007). Since activation of D1 receptors enhances neuronal excitability in the mPFC (Seamans and Yang, 2004), activation of these receptors may play a role in the increased burst firing of IL neurons Investigating the effect of D1 receptor antagonists on the extinction-related activity of IL neurons may define the exact role these receptors play during fear extinction. In the mPFC, D1- and D2-type receptors are expressed in both glutamatergic and GABAergic neurons (Vincent et al., 1993; Gaspar et al., 1995; Benes and Berretta, 2001). An important question is therefore how DA modulates activity in the IL microcircuitry to mediate consolidation of extinction memories. Future studies combining DA receptor pharmacology with cell-type-specific recordings of IL neuronal activity during extinction will be essential to address this question.

In line with findings in rodents, a recent study in humans shows that fMRI activity patterns observed in the vmPFC (the human analog of the IL) during extinction learning are reactivated shortly after extinction during memory consolidation and the number of these reactivations predicts extinction memory strength when tested the next day. Importantly, systemic administration of L-DOPA enhances vmPFC reactivations in parallel to improving extinction memory (Gerlicher et al., 2018) suggesting a critical role of DA in this process. Whether the vmPFC activity pattern reactivations in humans are related to burst firing of IL neurons observed in rodents during the consolidation of extinction memories and whether DA receptor activation in the IL/vmPFC mediates these extinction-related neuronal activity patterns in rodents and humans are open questions for further research.

While the studies summarized above highlight the crucial role DA plays in extinction memories, the source of DA release in the mPFC during fear extinction has not yet been identified. Anatomical studies demonstrate that the mPFC-projecting DA neurons are mainly located in the VTA (Lammel et al., 2008; Beier et al., 2015). Yet, the activity patterns of mPFC-projecting VTA DA neurons during consolidation and retrieval of extinction memories have remained elusive (Figure 1C). Interestingly, recent studies show that mPFC-projecting DA neurons are involved in encoding aversive events (Lammel et al., 2011, 2012; Vander Weele et al., 2018) and optogenetic activation of VTA DA terminals in the mPFC biases behavior toward aversion (Vander Weele et al., 2018). Further supporting the aversive nature of this DA input, optogenetic inhibition of VTA DA terminals in the IL enhances, rather than impairs, fear extinction (Luo et al., 2018). Together, these studies suggest that VTA DA input to mPFC is pro-aversive and therefore is unlikely to play a role in fear extinction. This raises the question of whether fear extinction is mediated by another source of DA input to mPFC. Supporting this idea, recent studies show that noradrenaline (NA) neurons located in the locus coeruleus (LC), in addition to NA, also co-release DA in the mPFC (Devoto et al., 2020). Furthermore, memory consolidation in the hippocampus is regulated by DA released from the LC, but not VTA, neurons (Kempadoo et al., 2016; Takeuchi et al., 2016). Therefore, the source of DA release in mPFC during fear extinction could be LC NA neurons (Figure 1C). Consistent with this possibility, LC projections to mPFC are indeed critical for the consolidation of extinction memories (Uematsu et al., 2017). Identifying the source of DA release in IL during fear extinction is an important outstanding question for further research.

## Concluding Remarks

Considerable progress has been made towards understanding the role DA plays in fear extinction. The studies reviewed above provide insights into how DA mediates different phases of extinction through its actions in the distinct components of the neural circuitry underlying fear extinction. The main conclusions and outstanding questions permitted from these studies are summarized in Figure 1. Overall, these findings highlight DA as a key regulator of fear extinction. An important implication of these findings is that enhancing DA signaling during extinction-based exposure therapy can be utilized as a therapeutic strategy in the treatment of anxiety disorders in humans. In line with this, systemic application of L-DOPA following fear extinction enhances extinction memories in humans (Haaker et al., 2013; Gerlicher et al., 2018); however, this enhancement depends on successful extinction learning (Gerlicher et al., 2019) indicating a boundary condition for facilitating the consolidation of extinction memories. Notably, in a genetic mouse strain with deficient extinction, systemic L-DOPA administration before extinction enhances extinction learning and memory (Whittle et al., 2016). Whether pre-extinction administration of L-DOPA can facilitate extinction learning in humans with deficient fear extinction and resistance to exposure therapy will be essential to investigate. Nevertheless, it is important to emphasize that DA is also a crucial mediator of fear learning and memory as well as aversive processing (Abraham et al., 2014; Lee et al., 2017; Likhtik and Johansen, 2019; Weele et al., 2019; Verharen et al., 2020), and hence systemic administration of pharmacological agents that enhance DA signaling globally in the brain can influence aversive processing and the strength of fear memories, as well. Caution is therefore required when systemically enhancing DA signaling. The development of more specific therapeutic approaches in the treatment of anxiety disorders would benefit from a deeper circuit-level understanding of how DA regulates different phases of fear extinction in distinct brain circuits.

## Author Contributions

XS-H and SD wrote the manuscript. All authors contributed to the article and approved the submitted version.

## Conflict of Interest

The authors declare that the research was conducted in the absence of any commercial or financial relationships that could be construed as a potential conflict of interest.
